# Supplementary material for: Causal Relationship between Aging and Anorexia Nervosa: A White-Matter-Microstructure-Mediated Mendelian Randomization Analysis
Source: Biomedicines. 2024 Aug 16;12(8):1874. doi: 10.3390/biomedicines12081874 (PMC11351342; doi:10.3390/biomedicines12081874)
Supplement: Supplementary file 1 [file biomedicines-12-01874-s001.zip › Supplementary Figure/Figure S4/Singe SNP plot of MR analysis for TL to ubm-b-2075.pdf]

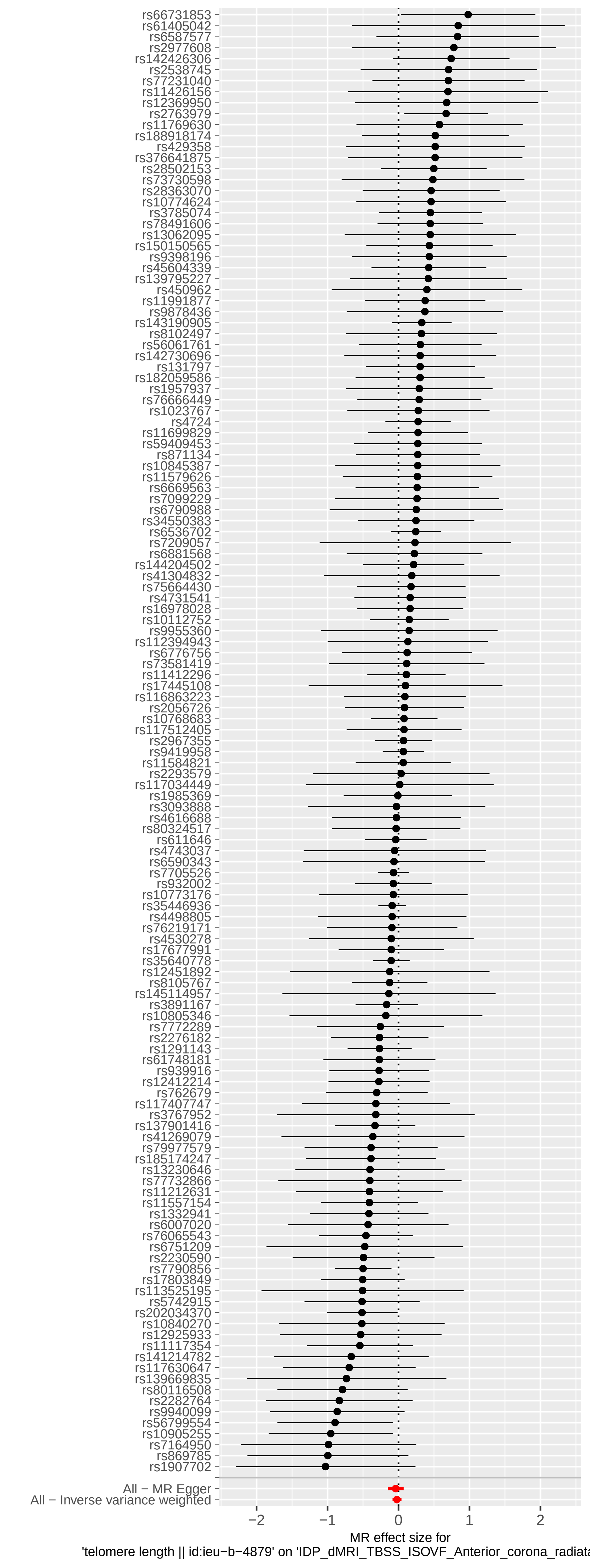
rs66731853
rs61405042
rs6587577
rs2977608
rs142426306
rs2538745
rs77231040
rs11426156
rs12369950
rs2763979
rs11769630
rs188918174
rs429358
rs376641875
rs28502153
rs73730598
rs28363070
rs10774624
rs3785074
rs78491606
rs13062095
rs150150565
rs9398196
rs45604339
rs139795227
rs450962
rs11991877
rs9878436
rs143190905
rs8102497
rs56061761
rs142730696
rs131797
rs182059586
rs1957937
rs76666449
rs1023767
rs4724
rs11699829
rs59409453
rs871134
rs10845387
rs11579626
rs6669563
rs7099229
rs6790988
rs34550383
rs6536702
rs7209057
rs6881568
rs144204502
rs41304832
rs75664430
rs4731541
rs16978028
rs10112752
rs9955360
rs112394943
rs6776756
rs73581419
rs11412296
rs17445108
rs116863223
rs2056726
rs10768683
rs117512405
rs2967355
rs9419958
rs11584821
rs2293579
rs117034449
rs1985369
rs3093888
rs4616688
rs80324517
rs611646
rs4743037
rs6590343
rs7705526
rs932002
rs10773176
rs35446936
rs4498805
rs76219171
rs4530278
rs17677991
rs35640778
rs12451892
rs8105767
rs145114957
rs3891167
rs10805346
rs7772289
rs2276182
rs1291143
rs61748181
rs939916
rs12412214
rs762679
rs117407747
rs3767952
rs137901416
rs41269079
rs79977579
rs185174247
rs13230646
rs77732866
rs11212631
rs11557154
rs1332941
rs6007020
rs76065543
rs6751209
rs2230590
rs7790856
rs17803849
rs113525195
rs5742915
rs202034370
rs10840270
rs12925933
rs11117354
rs141214782
rs117630647
rs139669835
rs80116508
rs2282764
rs9940099
rs56799554
rs10905255
rs7164950
rs869785
rs1907702
All − MR Egger
All − Inverse variance weighted
−2
−1
0
1
2
MR effect size for
'telomere length || id:ieu−b−4879' on 'IDP_dMRI_TBSS_ISOVF_Anterior_corona_radiata
